# “Slight” of Hand: The Processing of Visually Degraded Gestures with Speech

**DOI:** 10.1371/journal.pone.0042620

**Published:** 2012-08-09

**Authors:** Spencer D. Kelly, Bruce C. Hansen, David T. Clark

**Affiliations:** 1 Department of Psychology and Neuroscience Program, Colgate University, Hamilton, New York, United States of America; 2 Center for Language and Brain, Colgate University, Hamilton, New York, United States of America; Weill Cornell Medical College, United States of America

## Abstract

Co-speech hand gestures influence language comprehension. The present experiment explored what part of the visual processing system is optimized for processing these gestures. Participants viewed short video clips of speech and gestures (e.g., a person saying “chop” or “twist” while making a chopping gesture) and had to determine whether the two modalities were congruent or incongruent. Gesture videos were designed to stimulate the parvocellular or magnocellular visual pathways by filtering out low or high spatial frequencies (HSF versus LSF) at two levels of degradation severity (moderate and severe). Participants were less accurate and slower at processing gesture and speech at severe versus moderate levels of degradation. In addition, they were slower for LSF versus HSF stimuli, and this difference was most pronounced in the severely degraded condition. However, exploratory item analyses showed that the HSF advantage was modulated by the range of motion and amount of motion energy in each video. The results suggest that hand gestures exploit a wide range of spatial frequencies, and depending on what frequencies carry the most motion energy, parvocellular or magnocellular visual pathways are maximized to quickly and optimally extract meaning.

## Introduction

Speech and gesture are theorized to form an integrated system in language production [1], [2], and recent research has extended this claim to language comprehension [3]. However, little is known about what aspects of hand gesture are important for this integration. For example, it is not clear how much, or what type of, visual information is necessary for someone to extract meaning from gestures that accompany speech. In the current study, we used a standard spatial filtering technique to present visually degraded gestures with speech in order to determine the amount and type of signal in the visual processing system needed for successful processing of co-speech gestures.

Growing research in the past decade has demonstrated that gestures influence language comprehension (for a recent review, see [4]). By now, researchers understand a great deal about how, why and when gestures combine with speech during this process. For example, focusing on iconic gestures, which visually depict attributes and actions of objects and bodies in space (e.g., making a drinking gesture), we know that people integrate the meaning of gesture when processing the meaning of accompanying spoken utterance. This integration is so strong that some have recently argued that it is an obligatory [3] and even automatic process [5].

Importantly, all of this work assumes that co-speech gestures have some inherent and transparent meaning that people naturally and easily glean during language comprehension. In fact, one of the most salient features of co-speech gestures–in particular, iconic gestures–is that their form reflects their meaning in a *direct* and *non-arbitrary* fashion [2]. For example, the form and movement of different drinking gestures–a gentle movement with a small precision grasp depicts sipping from a sake cup whereas a more abrupt movement with a closed fist depicts drinking from a large beer mug–captures the meaning of these two actions in an obvious way. In contrast, spoken words reflect meaning only *indirectly* and *arbitrarily* through the particular conventions of a language. For example, the words, “nomu” in Japanese, and, “drink” in English, are utterly unrelated to the actual act of imbibing. This difference is exactly why co-speech gesture is so interesting–it offers a direct visual complement to the conventional symbols of a language, and when combined with those symbols, provides a more veridical “picture” of what a speaker means.

Although much progress has been made in understanding how people integrate the meaning of gesture and speech, a fundamental part of this process has been overlooked in the literature: how does the visual system process gesture in the first place? Or more specifically, which visual pathways are responsible for carrying information necessary to extract meaning from a co-speech gesture? The lack of attention to this basic question is striking in contrast to the rich tradition of psychophysical measurements of unimodal visual information. It is well known that the visual system employs a series of linear and non-linear transformations of the visual world that ultimately lead to an initial representation based on multiple spatial frequencies (see [6] for a review). This early representation of spatial frequencies is subserved by two primary visual pathways, namely the parvocellular (which in part relays high spatial frequencies, HSF) and magnocellular (which in part relays low spatial frequencies, LSF) pathways (e.g., [7], [8], [9]).

There is good evidence to suggest that visual information is processed at different rates along these two pathways. For example, LSF signals sent via the magnocellular pathway have been shown to reach visual cortex ∼10–20 ms before the HSF parvocellular signal [10], [11], [12], [13], and this advantage has been observed in human reaction time data for simplistic LSF stimuli [14], [15], [16], [17]. Furthermore, with respect to biological stimuli, people are more accurate and faster to discriminate human faces and recognize negative emotions using LSF vs. HSF visual information [18], [19], [20], [21].

Although this previous work has shown that LSF information is processed faster and more accurately than HSF information (at least when the stimuli are biological in nature), it is not clear how this extends to multimodal processing, such as the processing of gesture and speech. In fact, there are reasons to believe that *HSF information* may play a special role in this type of processing. First, from a unimodal standpoint it has been shown that both static and dynamic stimuli containing signs in American Sign Language (ASL) are more easily identified and more informative when filtered for HSFs (>1 cycle per degree, cpd, of visual angle) compared to LSFs [22], [23]. Next, consider the neuroanatomical finding that HSF information is primarily processed along the ventral processing stream (see [24], [25]), which leads directly to (among other areas) the superior temporal sulcus (STS), a region involved not only in language comprehension [26], but multimodal integration as well [27], [28].

In addition to this neuroanatomical evidence regarding the ventral visual processing stream, there is more direct functional evidence that HSF information is optimal for multimodal processing [29], [30]. For example, Munhall and colleagues [29] band-pass filtered videos of dynamic (i.e., mouths speaking) faces–containing different narrow bands of spatial frequencies–and asked participants to identify key words spoken in the videos. The main finding was that participants were better at identifying words when the video was in mid- to high-frequency bands compared to a speech-only baseline, whereas performance was no better than baseline in a low-frequency band. Moreover, Callan and colleagues [30] used fMRI to identify the STS as a possible mechanism for this effect. Thus, it appears that the multimodal regions in the brain are designed to optimally process mid- to high-frequency visual information when the stimuli consist of speaking faces.

Building on this previous research, the present study explores the multimodal processing of co-speech hand gestures during higher-level (semantic) processing of language. Indeed, in addition to being implicated in low-level phonemic processing, the STS is also known to process the meaning of language [26]. Moreover, the STS has been shown to process hand gestures that accompany speech [31], [32]. However, it is yet unknown how much, or what type of, visual information (in the form of spatial frequencies) is necessary for processing the meaning of gestures along with speech.

To explore this question, we presented visually degraded videos of a person gesturing while producing a semantically congruent or incongruent word. We degraded (i.e., filtered) the visual stimuli along two dimensions. In half of the degraded videos, we preserved low frequency information (LSF condition), and in the other half, we preserved high spatial frequency information (HSF condition). In addition, we had two filter bandwidths (i.e., two degradation levels), either moderate or severe. These four conditions were compared to a non-filtered baseline. The task was to identify whether the speech and gesture were congruent or incongruent.

If low spatial frequencies are optimal for gesture processing, participants should perform best in the LSF condition. In contrast, if high spatial frequencies are optimal, participants should perform best in the HSF condition. For both hypotheses, it is expected that the difference between LSF and HSF would be greatest in the maximally degraded condition.

## Results

### Prime-Target Congruence

Although participants were on average slower to respond to the incongruent items (M = 1167 ms, SE = 43 ms) compared to congruent items (M = 1136 ms, SE = 47 ms), F (1, 19) = 4.33, p = .05, η^2^ = .19, they made an equal number of errors (M = 0.07, SE = .008 and M = 0.06, SE = .006, respectively), F (1, 19) = 1.84, ns. Because error rates were comparable for incongruent and congruent items, all analyses below (including the exploratory item analysis) collapsed these two conditions.

### Error Rates

**Figure 1:**
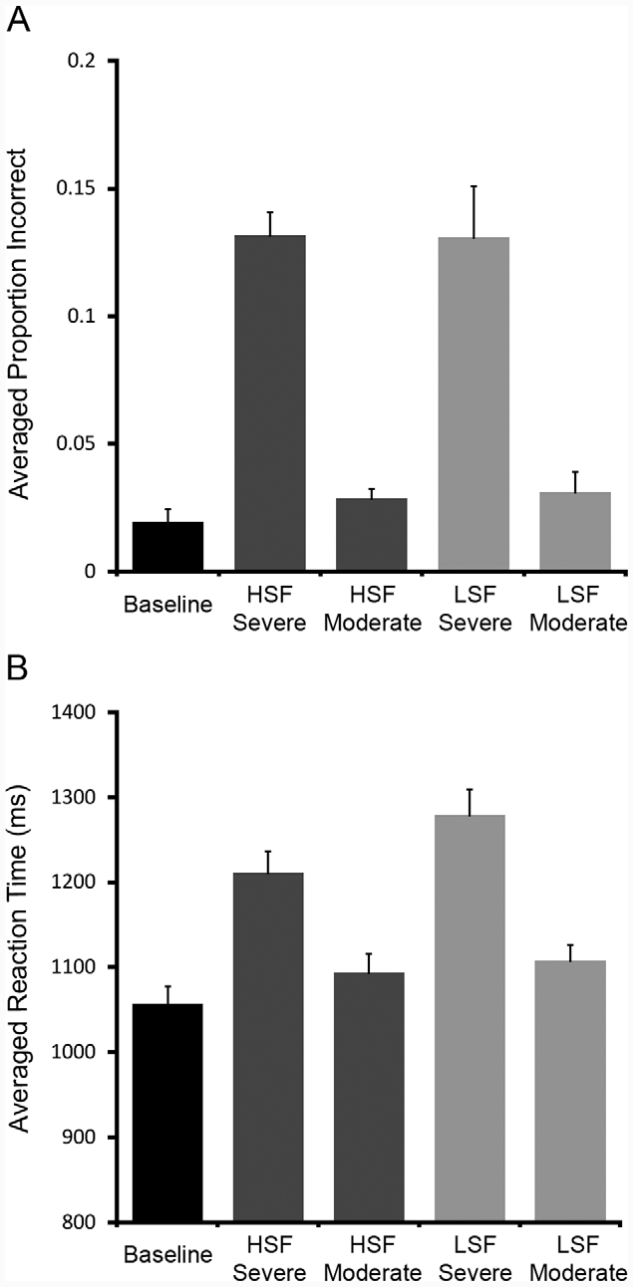
Error rates and response times for the five conditions. A) Error rates and B) response times.

There was a significant effect of video format, F (4, 76) = 29.12, p<.001, η^2^ = .60, with the Baseline video producing fewer errors than only the two severely degraded videos: HSF, tDS(4, 19) = 5.32, p<.001, and LSF, tDS(4, 19) = 10.10, p<.001 (see Figure 1A).

For the 2×2 analysis within the four filtered conditions, there was a significant main effect of degree F (1, 19) = 150.49, p<.001, η^2^ = .88, but not frequency F (1, 19) = 0.00, ns. Moreover, there was not a significant interaction of degree by frequency, F (1, 19) = 0.03, ns. Planned *t tests* showed that there were no significant differences between the HSF and LSF severely degraded videos, t(19) = 0.03, ns, or moderately degraded videos, t(19) = 0.27, ns. Thus, as Figure 1A illustrates, participants produced an equal number of errors for LSF and HSF stimuli in both the moderately degraded (∼3%) and severely degraded (∼13%) conditions, suggesting that both frequency filters provided approximately the same amount of visual information to participants.

### Response Times

There was a significant effect of video format, F (4, 76) = 30.08, p<.001, η^2^ = .61, with the Baseline condition producing faster reaction times than HSF severe, tDS(4, 19) = 4.92, p<.001, LSF severe, tDS(4, 19) = 8.68, p<.001, LSF moderate, tDS(4, 19) = 3.97, p<.001, but not the HSF moderate condition, tDS(4, 19) = 2.01, ns (see Figure 1B).

**Figure 2:**
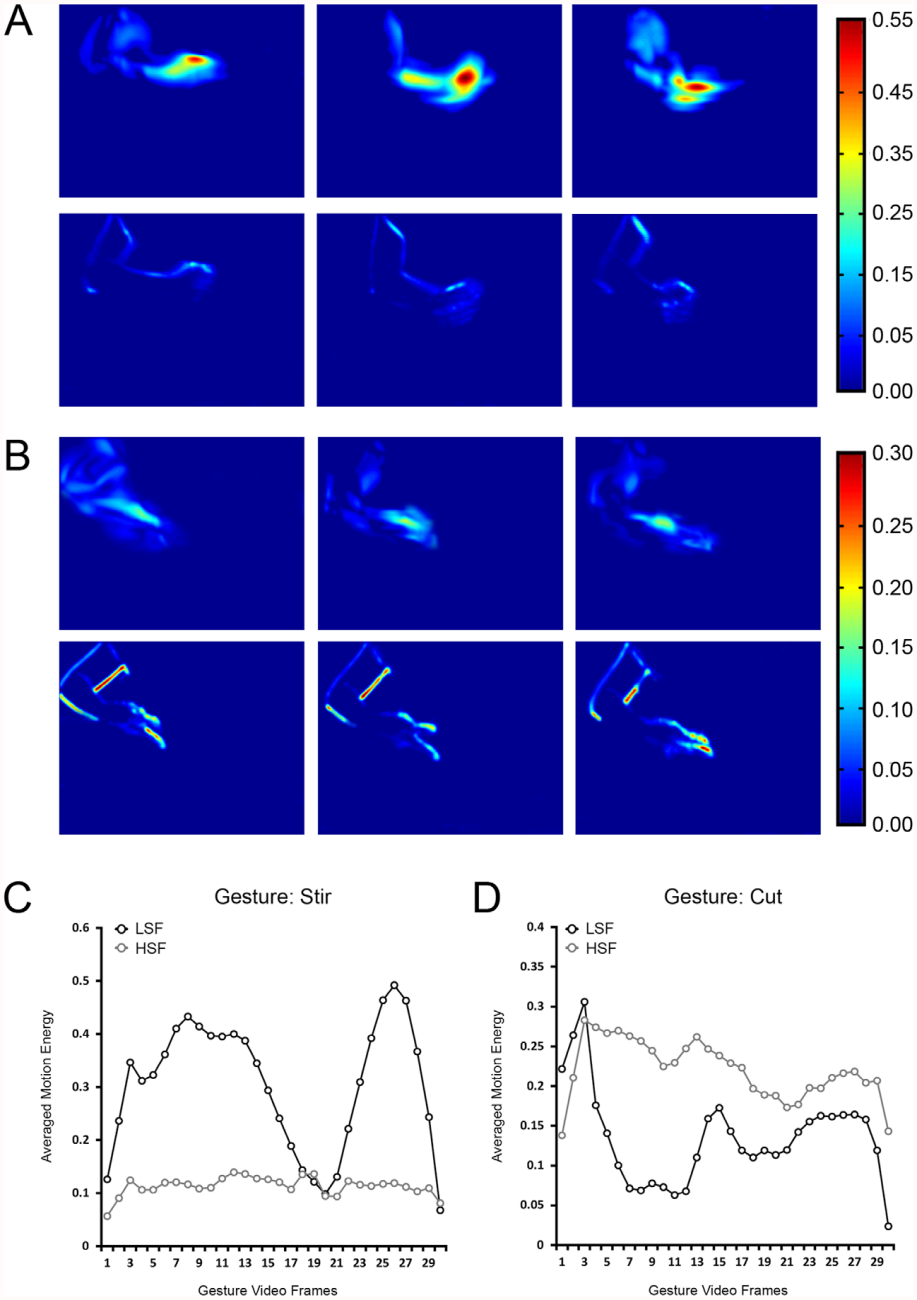
Motion energy analysis output for two gestures. (**A**) Motion energy for “stir” (a gesture with *faster* average RTs in the severely degraded LSF condition). The color bar to the far right shows motion energy values. Top row: LSF motion energy for the “stir” gesture video at three different time points in the video sequence. Bottom row: HSF motion energy for the “stir” gesture video at three different time points. (**B**) Same as (**A**), but for the “cut” gesture (a gesture with *slower* average RTs in the severely degraded LSF condition). Note the larger motion energy values for LSF in (**A**) and larger motion energy values for HSF in (**B**). (**C**) Averaged motion energy (ordinate) for the “stir” gesture video as a function of sequential frame in the gesture video (abscissa). Note the larger amount of motion energy for LSFs compared to HSFs. (**D**) Averaged motion energy (ordinate) for the “cut” gesture video as a function of sequential frame in the gesture video (abscissa). Note the larger amount of motion energy for HSFs compared to LSFs. Also note the scale differences in **A–D**.

For the 2×2 analysis within the four filtered conditions, there was a significant main effect of degree F (1, 19) = 76.30, p<.001, η^2^ = .80, in addition to frequency F (1, 19) = 5.68, p = .028, η^2^ = .23. And although there was not a significant interaction of degree by frequency, F (1, 19) = 2.39, p = .13, η^2^ = .10, planned *t tests* showed that within the severely degraded condition, the LSF condition produced significantly slower responses than the HSF condition, t(19) = 2.34, p = .03, η^2^ = .221 (two-tailed), whereas within the moderately degraded condition, there was no significant difference between the LSF and HSF moderate conditions, t(19) = 1.25, ns. See Figure 2b.

### Exploratory Item Analyses

Given the apparent HSF reaction time advantage reported above for the severely degraded condition, we sought to examine the robustness of such an advantage via exploratory analyses on our sixteen different gestures. We first sought to identify whether some gestures carry the RT advantage for HSFs in the severely degraded condition (since that is where the significant difference was found) more than others by subtracting (across participants) the mean HSF severe RT from the mean LSF severe RT on a gesture-by-gesture basis (16 items). Specifically, for a given gesture in the severely degraded condition, all participant reaction times (excluding outliers as described above) were averaged across congruent and incongruent trials in the LSF condition, and this was also done for the HSF condition. We then subtracted the averaged HSF severe RT from the averaged LSF severe RT (again, on a gesture-by-gesture basis). The result, shown in Table 1, revealed a very interesting categorical distinction (note that positive differences indicate an HSF advantage, while negative differences indicate an LSF advantage). That is, four of the gestures show a clear LSF RT advantage, whereas the other twelve show an HSF advantage, with the two sets separated by a 123.7 ms difference in RT. It is worth noting that comparable results are obtained when the item analysis described above is conducted on RTs from the congruent or incongruent trials separately, with a non-significant difference between the HSF-LSF RT differences for each gesture in the congruent and incongruent trials, t(15) = 0.698, p = 0.496.

**Table 1:** RT differences (LSF minus HSF) – Severely degraded condition.

Gesture	Mean Reaction Time Difference
Wipe	−240.72
Stir	−171.32
Saw	−114.40
Chop	−109.80
Shake	13.89
Twist	36.87
Scrub	76.51
Cut	128.56
Knock	152.54
Turn	166.57
Squeeze	172.44
Wring	177.38
Dial	178.76
Hammer	183.24
Slice	208.99
Type	223.38

Note: Gesture LSF-HSF RT differences have been sorted in ascending order.

#### Range-of-motion analysis

Upon subjective inspection of the four LSF advantaged gestures (compared to the other twelve HSF advantaged gestures), it became clear that our set of sixteen gestures involved a very broad range of movement across a significant area of the video screen. We therefore went about quantifying the range of movement by measuring how much space each gesture covered on the computer screen (in square centimeters). The range-of-motion for each gesture is given in Table S1. As expected from the subjective inspection, there was much variability in the amount of area that each gesture covered, from 1 cm^2^ to 16.5 cm^2^. Regressing the results of this range-of-motion analysis against the RT differences revealed a significant negative linear relationship (the smaller the range of motion, the larger the HSF RT advantage) that accounted for ∼59% (p<.001) of the variance in the RT differences. Thus, participants tended to show an LSF advantage when the range of movement was large, whereas they tended to show an HSF advantage when the range of movement was small. One way to think about such a relationship is that when gestures involve a small range of movement, the recognizability of the particular hand signals may possibly be restricted to fine-grained bursts of local movement that may be present when the gesture is restricted to a narrow band of HSFs, but would likely be obscured if the gesture was restricted to a narrow band of LSFs. However, when the range of movement is large, the gesture signal may be spread out across a large region of space. Because LSFs are restricted to large-scale coarse representations, they may be optimally suited to process gestures involving broad-sweeping movement.

#### Motion energy analysis

While the above account is appealing, it is largely speculative as the range-of-motion analysis is not specific to any given band of spatial frequencies (i.e., identical estimates of motion area would be produced in the LSF or HSF filtered videos). Further, it is possible that the range of motion of a gesture does not necessarily correlate with what spatial frequency carries the most informative signal for identifying that gesture. For example, Table S1 shows that “hammer” has the fourth largest range of motion, but it is possible that the fine movements of the hand (HSF) are more visually informative than the broad movements of the arm (LSF). Thus, while the range-of-motion analysis strongly suggests a relationship between the amount of space covered by a gesture and the subsequent recognition of that gesture, it is not specific to any particular band of spatial frequencies.

Given the limitation of the range-of-motion analysis described above, we sought to investigate whether the extent of the motion signal contained in either the LSF or HSF bands could account for the RT differences reported in Table 1. In order to achieve this, we employed a motion energy analysis inspired by standard spatiotemporal motion energy models designed to reflect how visual information is processed in early visual cortical areas (e.g., [33], [34], [35]). Specifically, motion energy models employ spatial filters similar to those used in the current experiment to assess motion “detectability” (i.e., the extent to which reliable motion can be perceived by an observer) of an object across time. And, since motion energy models employ spatial filters that can be set to any particular spatial band, they can be used to estimate motion perceptibility within different bands of spatial frequency. Thus, within the confines of the current study, a motion energy analysis would allow us to assess the extent to which the speed of co-speech gesture recognition depends on the amount of motion energy available in a given range of spatial frequencies.

To explore this, we subjected all *non-filtered gesture videos* (i.e., videos from the baseline condition) to a spatiotemporal motion energy analysis based on that reported by [33]. The details behind the implementation of the analysis can be found in a report by [36] (without the saliency component). Briefly, the basis of a spatiotemporal energy model stems from the fact that the motion of any given object can be represented as a single pattern plotted in a 3-dimensional (3D) x-y-t space, such that the position of a given piece of an object is tracked across space (horizontally via x and vertically via y) as a function of time (t). Specifically, with respect to the gesture videos, an x-y-t volume consists of either a “stack” of 2-dimensional (2D) x-t slices taken from the same *row* of pixels across all frames (i.e., horizontal motion), or a “stack” of 2D y-t slices taken from the same *column* of pixels across all frames, transposed and stacked vertically (i.e., vertical motion). In either of these stacks, motion is characterized by slanted traces, with the slope of the traces being proportional to velocity [33], [36]. Thus, motion energy within a given row (or column) of pixels across time can be measured by differencing (is this an accepted verb?) the output of spatiotemporally oriented filters row-by-row (or column by column) across all frames for objects within any given video sequence [36]. The spatiotemporal filters used in the current analysis are exactly those reported in previous models (e.g., [33], [36]), with the exception that motion energy was calculated separately for LSFs or HSFs (with the filters tuned to a 1-octave bandwidth as was used in the severely degraded condition). That is, each non-filtered gesture video was filtered twice, once to extract motion energy in the LSF band, and again to extract motion energy from the HSF band. The extraction of motion energy at the two different spatial frequency bands was achieved by scaling the spatiotemporal filters to match the spatial frequency bands in either the LSF or HSF conditions of the behavioral experiments reported above. Refer to Figure 2 for an example of a gesture with the majority of motion energy in the LSF band (panel A) and HSF band (panel B). Figures 2C and 2D provide a frame-by-frame graphical illustration of the relative motion energy advantage for an LSF gesture (stir) and HSF gesture (cut). Frame-by-frame graphical illustrations of motion energy for each gesture used in the current study are given in Figure S1.

**Figure 3:**
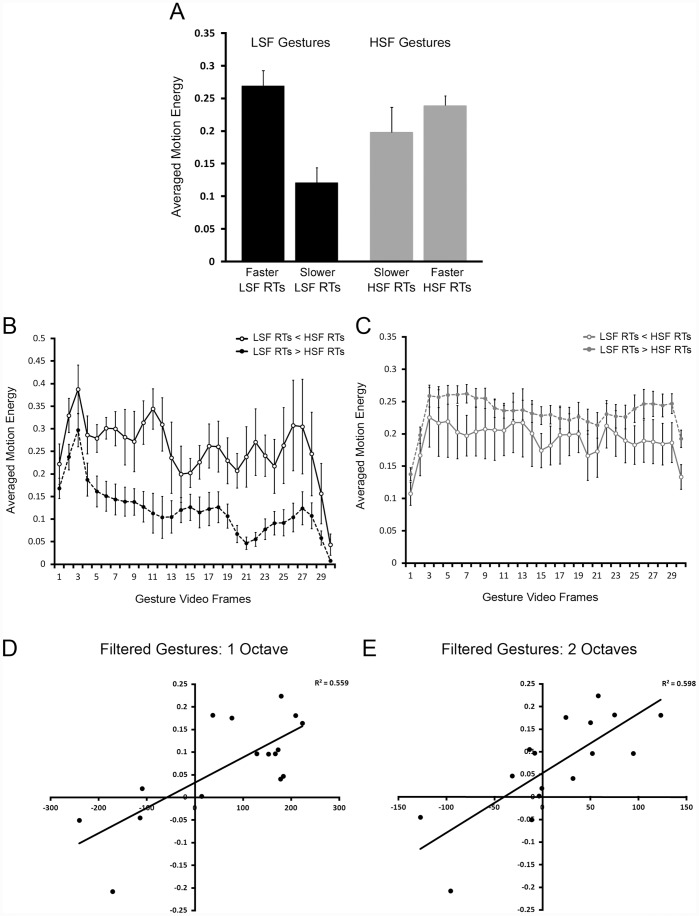
Motion energy analysis output for all gesture videos. (**A**) Averaged motion energy output for LSF and HSF gesture videos grouped by RT differences as reported in Table 1 (i.e., top 4 vs. bottom 12). That is, the left black bar and the left gray bar show the averaged motion energy when RTs were faster in the LSF condition (n = 4), and the right black bar and the right gray bar show the averaged motion energy when RTs were faster in the HSF condition (n = 12). Not that the faster RTs in the LSF condition occurred when there was a high amount of LSF motion energy. Error bars are ±1 S.E.M. (**B**) and (**C**) Show averaged motion energy (ordinates) across all frames in the gesture videos (abscissas). (**B**) Shows averaged motion energy in the LSF filtered gesture videos yielding *faster* RTs (open circles, solid black trace), and averaged motion energy in the LSF filtered gesture videos yielding *slower* RTs (solid circles, dashed black trace). Note the larger amount of motion energy for the solid black trace with open circles. (**C**) Shows averaged motion energy in the HSF filtered gesture videos yielding *slower* RTs (open circles, solid gray trace), and averaged motion energy in the HSF filtered gesture videos yielding *faster* RTs (solid circles, dashed gray trace). Note the larger amount of motion energy for the dashed gray trace with solid circles. (**D**) Relationship between RT differences in the 1 octave filtered condition (LSF RTs minus HSF RTs; abscissa) and motion energy differences (HSF motion energy minus LSF motion energy; ordinate). While the RT difference is clearly a categorical one, motion energy accounts for ∼55.9% of the variance in RT differences for that condition (p<0.001). (**E**) Relationship between RT differences in the 2 octave filtered condition (LSF RTs minus HSF RTs; abscissa) and motion energy differences (HSF motion energy minus LSF motion energy; ordinate). Motion energy accounts for ∼59.8% of the variance in RT differences for that condition (p<0.001).

Next, we divided our videos into two groups, one in which RTs were faster for the severely degraded LSF vs. HSF conditions (top four gestures listed in Table 1) and the other in which RTs were faster for the severely degraded HSF vs. LSF conditions (bottom twelve gestures listed in Table 1). We then calculated the average motion energy for each gesture in each group by averaging motion energy within each frame, and then taking the mean across all frames in a given gesture video sequence and averaging across all gesture videos in each group (refer to Figure 3A for further details). We also conducted an average motion energy analysis (for either LSFs or HSFs) by averaging motion energy across videos in a given group on a frame-by-frame basis (refer to Figure 3B and 3C for further details).

While Figure 3A–C yield a nice illustration of the difference in motion energy between the gestures grouped according to Table 1, they cannot by themselves provide meaningful explanatory power given the large difference in sample size (e.g., n = 4 vs. n = 12). We therefore ran a regression between spatial frequency advantage (HSF RTs minus LSF RTs, collapsed across congruent and incongruent trials) and amount of motion energy difference (HSF motion energy minus LSF motion energy) for the 1 octave (severe) and 2 octave (moderate) conditions (refer to Figure 3D and 3E respectively). Note that the motion energy differences were calculated by averaging motion energy across all frames for each gesture (separately for LSF and HSF motion energy). The averaged LSF and HSF motion energy values are illustrated in Figure S2 (with gestures sorted according to Table 1). Although the RT difference is clearly a categorical one, motion energy accounts for ∼55.9% of the variance in RT differences for the severely degraded condition (p<0.001) (Figure 3D) and ∼59.8% of the variance in RT differences for the moderately degraded condition (p<0.001) (Figure 3E). This finding therefore suggests that a substantial portion of the variance of the RT advantage is explained by the extent to which motion energy is concentrated in either the LSF or HSF band.

## Discussion

The results provide support for both of our predictions. Although there was an overall HSF advantage for response times, this effect was modulated by the range of movement and amount of motion energy in the LSF and HSF videos. That is, co-speech gestures exhibiting a large range of motion tended to elicit an LSF advantage, while co-speech gestures exhibiting a narrow range of motion tended to yield an HSF advantage. However, and crucial to the current study, the spatial frequency advantage observed here tended to co-vary with the spatial frequency band that captured the majority of the motion energy signal. Thus, the range of spatial frequencies important for co-speech gesture recognition seems to depend on which spatial frequency band contains the larger portion of motion energy.

Considering the high level of degradation in both of the severely degraded video conditions, it is remarkable that participants were able to do our task at all. In fact, they did it quite well (i.e., performance was far above chance in all conditions). The ability to successfully relate even severely degraded gestures to spoken words lends credence to recent claims that gestures have a deep connection to speech during language comprehension [3], [5].

Although we found that certain gestures offered an HSF advantage and others offered a LSF advantage, this variability is actually consistent with the previous literature. For example, the findings of an HSF advantage are consistent with research demonstrating that mid- to high- frequencies are optimal for processing lip movements [29], [30]. As with lip movements, the twelve gestures in the present study that demonstrated an HSF RT advantage exploited a very small range of motion and packed most of their motion energy into high frequencies. To illustrate, consider our “slicing” gesture. To correctly understand that gesture’s meaning, one has to process mainly the fine-grained movements of one hand (a closed fist making small back-and-forth cutting movements). Because most of the important information needed to understand this slicing gesture resides in HSF bands, it makes sense that participants would be faster to process it when stripped down (filtered) to contain only HSF information.

In contrast, for the four gestures that covered a wide range of motion and packed most of their motion energy into the LSF band, there was an RT advantage for our LSF filtered condition. This finding is interesting in light of research showing that people are faster to discriminate human faces and recognize negative emotions using LSF vs. HSF visual information [18], [19], [20], [21]. Although faces clearly have many fine-grained details, it is well established that face discrimination relies primarily on global and holistic visual information [18], [19], precisely the sort of information that is ideally suited for LSF processing. Together, these findings suggest that for gestures packing most of their motion energy into very fine-grained movements (e.g., like dialing and slicing), HSF bands are optimal for processing meaning; whereas for gestures placing most of their motion energy into very coarse-grained movements (e.g., like stirring and sawing), LSF bands are optimal.

The present study makes a novel contribution to the neuroscience of multimodal speech processing. For example, although previous work has manipulated the clarity of speech to investigate the role of co-speech gesture on language comprehension [37], [38], to our knowledge, no study has done the opposite. By degrading our videos along spatial frequency, we were able to explore questions not previously addressed in the literature. Specifically, by filtering the gesture videos to contain only LSF or HSF visual information, we were able to band gestures along two early visual pathways, namely the magnocellular pathway for LSF stimuli and parvocellular pathway for HSF stimuli (see [24]). This allowed us to address not only how much spatial frequency content (in the form of level of degradation, i.e., octave bandwidth) each band needed before it could be processed, but also which pathway may play a greater role in the processing of gestures that accompany speech. Based on the results reported in the current study, it appears that: 1) regardless of which visual pathway relays the gesture information, successful processing can occur even with limited spatial frequency content (as noted above, error rates in all conditions were well above chance), suggesting that the processing of gesture may not require a full range of spatial frequencies, and as mentioned earlier, may take place during the early stages of visual analysis; and 2) it appears that different visual pathways may be activated depending on whether gestures place the majority of motion energy in either low or high frequency bands.

These results also have implications for theories of gesture-speech integration [1], [2], [39]. It is now well established that listeners (viewers) glean meaning from co-speech gesture, and this information significantly impacts the semantic processing of speech during language comprehension (for reviews, see [4], [40]). However, this previous research has taken for granted that the visual system has done extensive processing to assemble meaning from these gestures in the first place. The present study took an important step back and attempted to describe this early stage of gesture processing according to basic neuroanatomy of the visual system. By showing that different gestures carry different frequencies that optimally exploit different visual pathways (with respect to the motion energy signal), it is clear that not all gestures are created equal during visual processing–some exploit high frequencies, some exploit low frequencies and some exploit both. These results are a first step to better understanding the psychophysical mechanisms by which low-level visual information from gesture is combined with low-level auditory information from speech to create meaning during language comprehension. Indeed, it would be interesting for future work to explore whether particular gestural frequencies are optimized to interact with particular speech frequencies at the earliest stages of gesture-speech integration in multimodal processing sites in the brain (e.g., superior temporal sulcus, inferior parietal lobule, and inferior frontal gyrus). By taking such a low-level psychophysical approach to describing gesture-speech integration, gesture researchers–who mostly come from a language background–will hopefully come to see this integration process as part of a larger “binding problem,” a problem that has rich traditions of research in other well-established disciplines (e.g., visual science and computational neurobiology).

**Figure 4:**
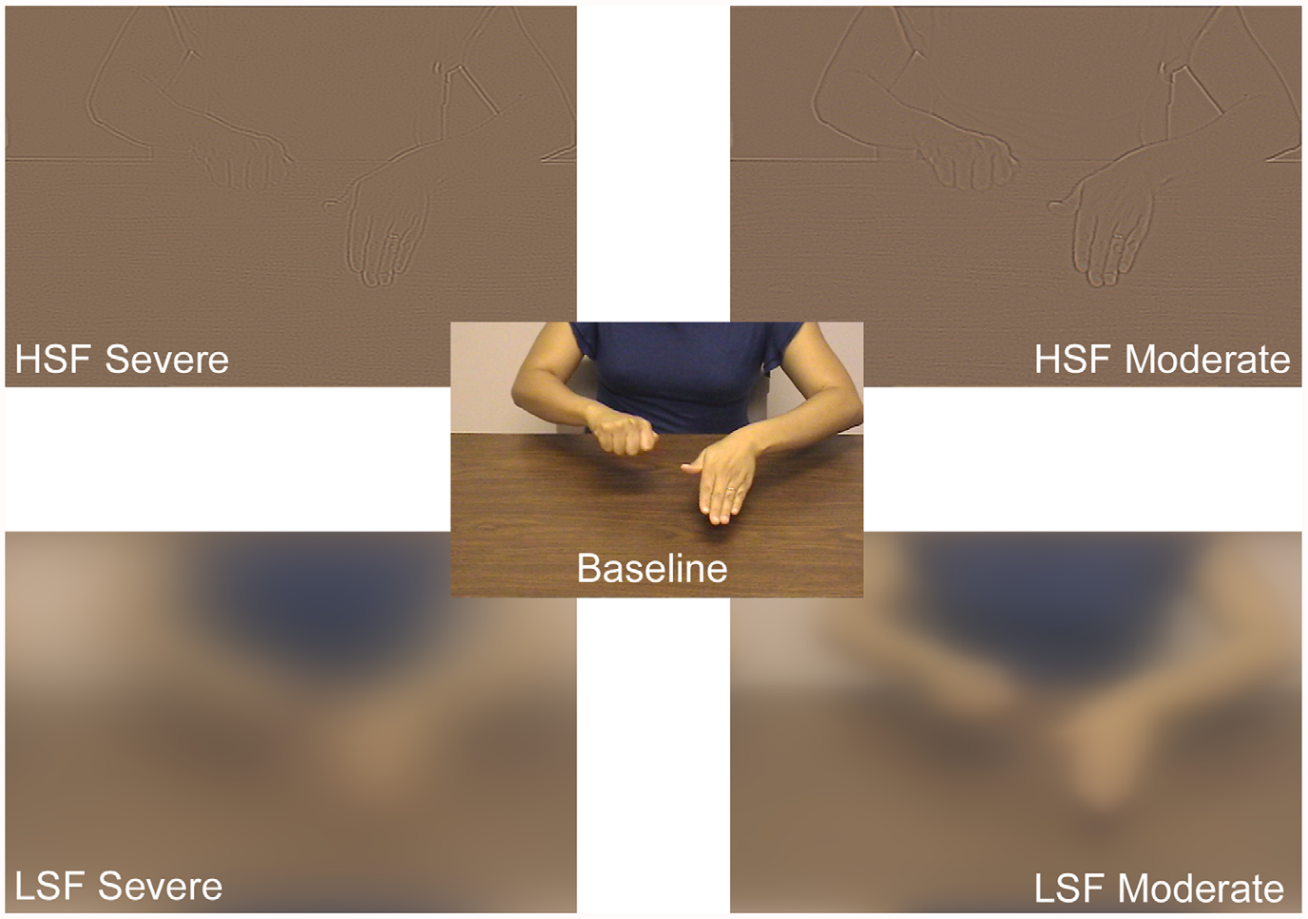
A single frame from the Baseline and four visually degraded videos. The example shows a congruent stimulus (speech: “chop”), but the video would be identical for an incongruent stimulus, with the only difference being that the speech was “twist.”

## Methods

### Ethics Statement

The study was approved by the university’s Institutional Review Board (IRB). Prior to the experiment, all participants read and signed an informed consent form that was also approved by the IRB.

### Participants

Twenty right-handed college undergraduates (10 female) participated in this experiment as part of the university’s Introduction to Psychology course. All were native English speakers and had normal (or corrected to normal) vision.

### Materials

The stimuli were 1-s videos of the torso of a female actor making a gesture and simultaneously saying a verb (adapted from [3]). Half the videos presented the same information through gesture and speech (e.g., saying and gesturing “chop”), and the other half presented different information (e.g., saying “twist” and gesturing chop).

Stimuli for five conditions were created using an in-house program in MATLAB 2008a equipped with the Image Processing Toolbox (ver 6.1) and Signal Processing Toolbox (ver 6.9). One condition was not visually filtered and was used as a baseline. The remaining four videos were filtered along two dimensions. The first dimension was spatial frequency, with one level centered on a high spatial frequency (11 cpd) and the other on a low spatial frequency (0.25 cpd). The second dimension was level of degradation, with one filtered to contain a 1 octave bandwidth (severely degraded) and the other, a 2 octave bandwidth (moderately degraded) (see Figure 4). The specific design and parameters of the filters can be found in [41]. The visual angle of the videos (width) was 9° (viewed from 1.168 m). All videos were viewed on a monitor with a pixel resolution of 1400×900.

In total, there were 80 congruent videos (5 versions of 16 different items) and 80 incongruent videos. The order of all 160 trials was randomized across participants.

### Procedure

Participants were instructed to press one keyboard button (“yes”) if the gesture and speech contained congruent information and a different button (“no”) if they contained incongruent information. Although explicitly directing attention to the relationship between gesture and speech is not how people typically process multimodal language in everyday interactions, we chose this task because it is the most straight-forward first step to testing how much and what type of visual information is optimal for extracting meaning from gestures.

Error rates (proportion incorrect) and response times (milliseconds) were analyzed from the onset of the congruent and incongruent trials to determine how well participants could evaluate the semantic relationship between the gesture and speech in each video. Response times that were more than two standard deviations from the mean were excluded.

### Design and Analysis

First, we compared the mean error rates and response times of the five congruent to the five incongruent trials. Based on this analysis, we collapsed congruent and incongruent trials into a single score for each of the five conditions. Following this, we ran a one-way repeated measures ANOVA (baseline, HSF moderate, HSF severe, LSF moderate, LSF Severe) comparing the baseline condition to the four filtered conditions. Finally, excluding the baseline condition, we computed a 2 (frequency: HSF and LSF) by 2 (degree: moderate and severe) repeated-measures ANOVA on the error rates and RTs for only the four filtered stimuli. Because we had a priori predictions regarding differences between low and high spatial frequencies, we computed planned orthogonal *t tests* comparing these two conditions within the moderate and severe conditions regardless of whether there was an omnibus interaction (see [42]).

Following from the results yielded in the main analysis mentioned above, we carried out an exploratory gesture item analysis based on response time (RT) differences between the HSF and LSF filtered gesture video conditions. The results of the item analysis were regressed against a physical analysis of the range-of-motion (further described in the *Results* section) for each gesture video (un-filtered). The findings from the range-of-motion analysis motivated us to subsequently explore the role of motion cues in the different spatial frequency bands with a standard motion energy analysis (further explained in the *Results* section).

### Conclusion

In conclusion, we have for the first time demonstrated that hand gestures exploit a wide range of spatial frequencies, and depending on what frequency carries the most motion energy, different visual pathways (i.e., parvocellular and magnocellular) are likely maximized to quickly and optimally extract meaning. This novel finding not only provides insights into the type and amount of visual information necessary to process hand gestures during early stages of visual processing, but it also represents an important step to better understanding how people visually process complex and dynamic multimodal information in face-to-face communicative contexts.

## Supporting Information

Figure S1 **Frame-by-frame graphical illustrations of motion energy for each of the 16 gestures.** Averaged motion energy is plotted on the ordinate, and sequential frames in the videos are plotted on the abscissa.
(TIF)

Figure S2 **Averaged LSF and HSF motion energy values for each of the 16 gestures.** They are sorted according to Table 1.
(TIF)

Table S1 Range of motion values (cm^2^) for each of the 16 gestures.
(DOCX)
