# Supplementary figures and images for: “Slight” of Hand: The Processing of Visually Degraded Gestures with Speech
Source: PLoS One. 2012 Aug 9;7(8):e42620. doi: 10.1371/journal.pone.0042620 (PMC3415388; doi:10.1371/journal.pone.0042620)

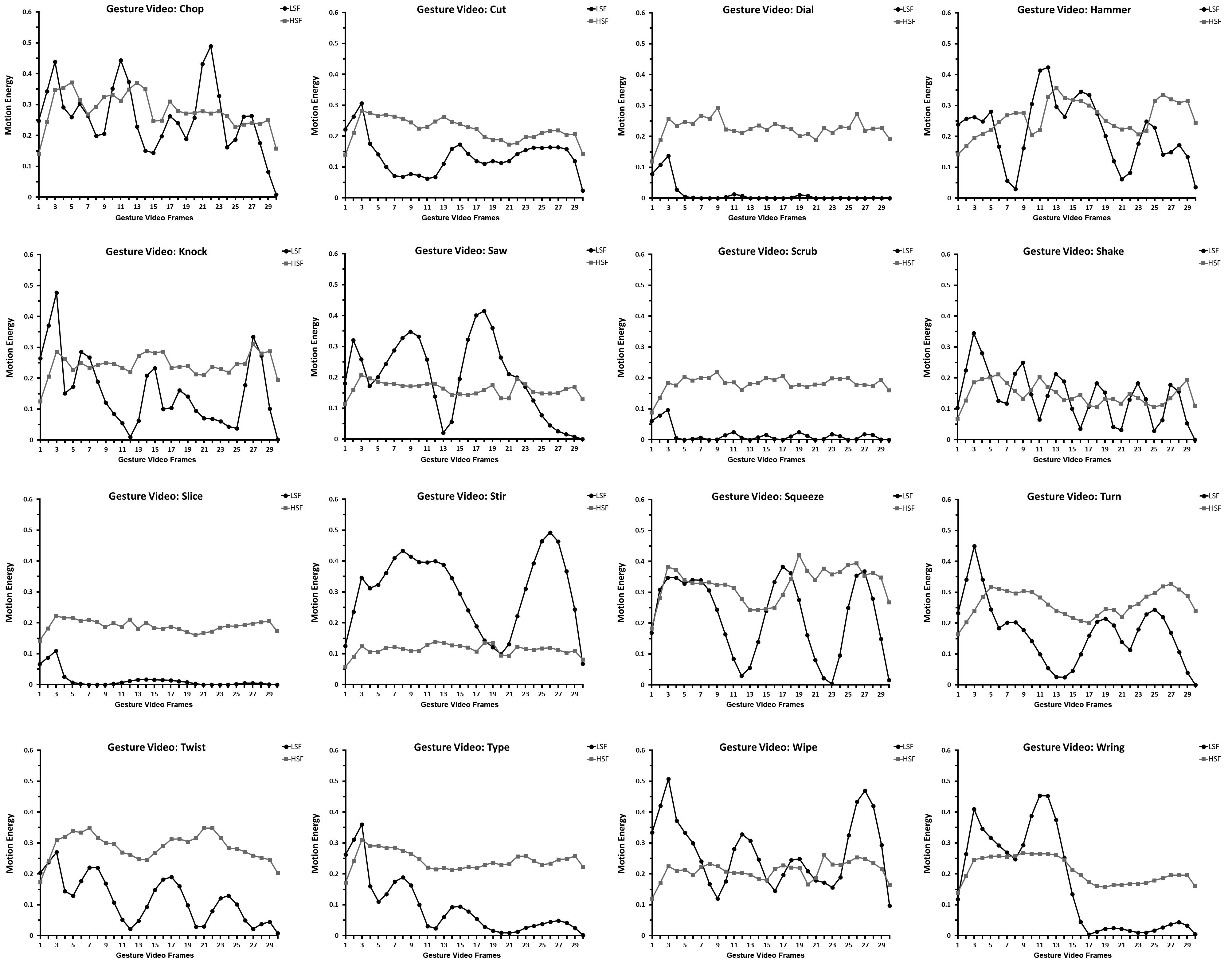

Supplement: Figure S1 — Frame-by-frame graphical illustrations of motion energy for each of the 16 gestures. Averaged motion energy is plotted on the ordinate, and sequential frames in the videos are plotted on the abscissa. (TIF) [file pone.0042620.s001.tif]

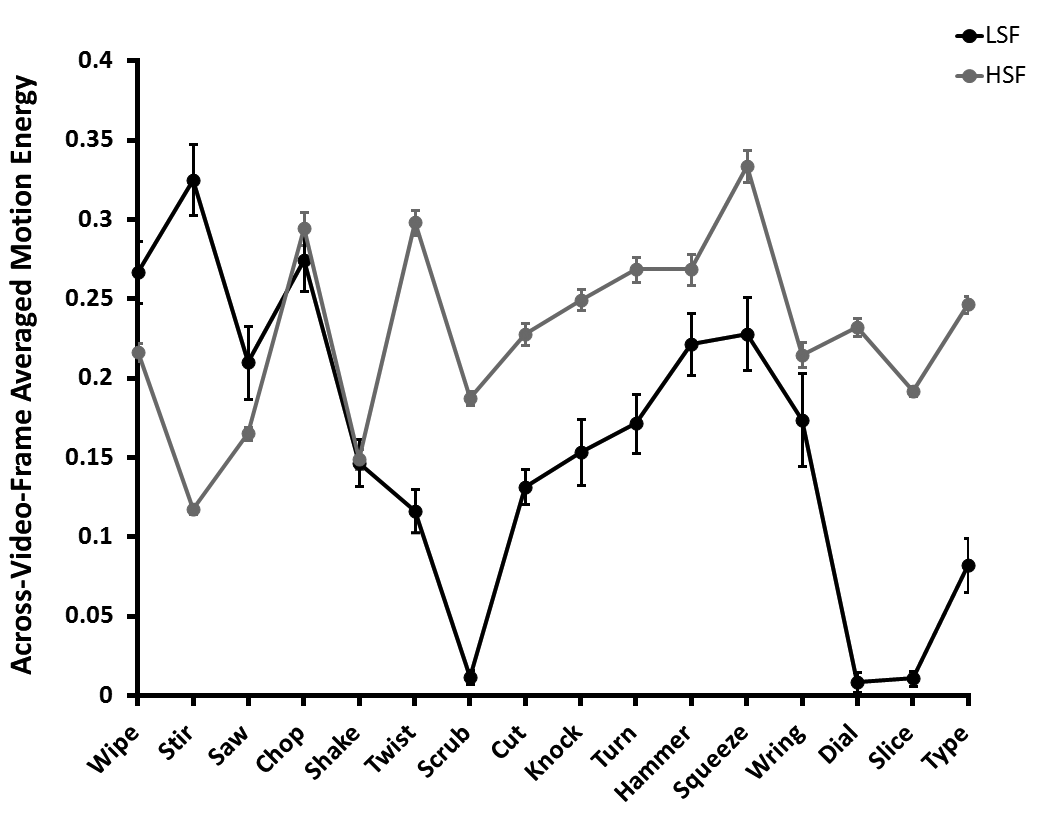

Supplement: Figure S2 — Averaged LSF and HSF motion energy values for each of the 16 gestures. They are sorted according to Table 1. (TIF) [file pone.0042620.s002.tif]
