# Supplementary material for: “Slight” of Hand: The Processing of Visually Degraded Gestures with Speech
Source: PLoS One. 2012 Aug 9;7(8):e42620. doi: 10.1371/journal.pone.0042620 (PMC3415388; doi:10.1371/journal.pone.0042620)
Supplement: Table S1 — Range of motion values (cm2) for each of the 16 gestures. (DOCX) [file pone.0042620.s003.docx]

**Supplementary Table 1.** Range of motion values by gesture item

| **Gesture** | **Range of Motion (cm^2^)** |
| --- | --- |
| Twist | 1 |
| Type | 1 |
| Turn | 1.5 |
| Dial | 2.25 |
| Knock | 2.5 |
| Scrub | 2.5 |
| Slice | 2.5 |
| Cut | 3.5 |
| Shake | 4.5 |
| Saw | 6 |
| Squeeze | 6 |
| Wring | 6 |
| Hammer | 8 |
| Chop | 8.75 |
| Stir | 15 |
| Wipe | 16.5 |

Note: These items have been sorted in ascending order from lowest to highest range of motion
